# Effects of enteral immunonutrition on postoperative complications of colorectal cancer surgery: a systematic review and meta-analysis of randomized controlled trials

**DOI:** 10.3389/fnut.2026.1861667

**Published:** 2026-06-22

**Authors:** Yuhang Wu, Qingyu Du, Chenglin Zhao, Guo Zu

**Affiliations:** 1 Department of Gastroenterology Surgery, Central Hospital of Dalian University of Technology (Dalian Municipal Central Hospital), Dalian, China; 2 Department of Graduate School, Dalian Medical University, Dalian, China

**Keywords:** colorectal cancer surgery, enteral immunonutrition, meta-analysis, postoperative complication, randomized controlled trials

## Abstract

**Objective:**

Enteral immunonutrition is extensively administered to patients who have undergone colorectal cancer (CRC) surgery. However, its efficacy in reducing postoperative complications and the length of hospital stay (LOS) remains controversial. The aims of our meta-analysis were to investigate the effects of enteral immunonutrition on postoperative complications in patients undergoing CRC surgery.

**Methods:**

We searched the PubMed, Web of Science, and Wanfang databases from January 2000 to December 2025 and the reference lists of selected randomized controlled trials (RCT) The odds ratio (OR), weighted mean difference (WMD) and 95% confidence interval (CI) were used to assess the association between enteral immunonutrition and postoperative complications in patients undergoing CRC surgery.

**Results:**

Nine studies involving 1,198 patients with CRC were identified. Our results demonstrated that enteral immunonutrition could reduce the incidence of infectious complications 95% CI among postoperative CRC patients (OR: 0.48; 95% CI: 0.34–0.66; *P* < 0.001). The results of the subgroup analysis revealed that both preoperative enteral immunonutrition and postoperative enteral immunonutrition could reduce the incidence of postoperative infectious complications in CRC patients. Furthermore, our results revealed that enteral immunonutrition was not associated with postoperative anastomotic leakage (OR: 0.56; 95% CI: 0.31–1.03; *P* = 0.063), ileus (OR: 0.74; 95% CI: 0.44–1.24; *P* = 0.249), or LOS (WMD = −0.78; 95% CI: −2.02 to 0.47; *P* = 0.222), and we found no associations between enteral immunonutrition and anastomotic leakage, ileus and LOS in patients who underwent CRC surgery.

**Conclusions:**

Enteral immunonutrition could reduce the incidence of infectious complications in patients undergoing CRC surgery. However, it did not reduce the incidence of postoperative anastomotic leakage or ileus and did not shorten LOS of patients who underwent CRC surgery.

**Research registration unique identifying number (UIN):**

The study protocol was registered in the International Prospective Register of Systematic Reviews (PROSPERO) under registration number CRD420261288947.

**Systematic review registration:**

https://www.crd.york.ac.uk/PROSPERO/view/CRD420261288947, identifier: CRD420261288947.

## Introduction

Colorectal cancer (CRC) has emerged as one of the most prevalent malignant tumors worldwide (1). The incidence rate has been steadily rising annually, with a striking trend toward earlier onset observed over the past few decades, posing a significant public health challenge (2, 3). Currently, surgical intervention remains the cornerstone of curative treatment for CRC, serving as the universally recognized “gold standard,” as endorsed by oncology guidelines and esteemed medical bodies such as the American Society of Clinical Oncology (ASCO) (4). However, surgery significantly compromises the physical condition of CRC patients, due to its characteristics including trauma and bleeding, and many patients suffer from malnutrition, which can cause many complications and greatly affect their prognosis (5). The most common post-surgical complications of CRC include infections, anastomotic leakage, ileus, etc. (6, 7). These postoperative complications can significantly prolong patient hospitalization and impose a heavy financial burden.

Therefore, it is highly important to search for methods that can lower the incidence of complications and improve prognosis. For example, some scholars have shown that the preoperative correction of anemia (8), the administration of antibacterial agents (9), and other measures can reduce postoperative complications in patients with CRC. In recent years, the effects of perioperative immunonutritional preparations on postoperative complications in CRC patients have attracted increasing attention. Immunonutritional preparations, as a potential solution, have received extensive attention, with compositions that integrate amino acids (glutamine and/or arginine), polyunsaturated fatty acids (omega-3 fatty acids), and nucleotide or RNA mixtures administered via enteral or parenteral routes. While several randomized controlled trials (RCTs) have explored their benefits in CRC patients (10, 11), the results remain inconsistent. For example, Finco et al. (12) reported that enteral immunonutrition could not lower the rate of postoperative infectious complications; by contrast, Braga et al. (13) reported that preoperative enteral immunonutrition could significantly lower the rate of infectious complications, but postoperative enteral immunonutrition could not lower the rate of infectious complications. Therefore, our meta-analysis aimed to investigate the effects of enteral immunonutrition on postoperative complications in CRC patients.

## Materials and methods

### Search strategy

This meta-analysis was conducted on the basis of a comprehensive literature search across multiple databases, including the PubMed, Web of Science, Scoupus Preview, Elsevier ScienceDirect, CNKI and Wanfang databases, spanning from January 2000 to December 2025; the final search date was April 5, 2026. The search was performed using a combination of medical subject headings (MeSH) and free-text terms, including “colorectal neoplasms,” “colorectal cancer,” “surgery,” “immunonutrition,” “complication,” and “randomized controlled trials” or “RCT.” The search strategy was designed to identify all relevant studies evaluating the impact of preoperative immunonutrition on postoperative outcomes in patients who underwent CRC surgery.

Methodologically rigorous and transparent study selection was performed in accordance with the PRISMA (Preferred Reporting Items for Systematic Reviews and Meta-Analyses) guidelines for systematic reviews and meta-analyses. The screening workflow followed the Population, Intervention, Comparison, Outcome, Study Design (PICOS) criteria, with inclusion limited to studies involving adult patients undergoing elective CRC surgery, receiving enteral immunonutrition intervention, and reporting postoperative complications as outcomes. To maximize the sensitivity of the evidence synthesis and include all available data, only RCTs were selected, regardless of the language in which they were published.

All retrieved records underwent relevance screening based on titles and abstracts, followed by full-text evaluation to determine eligibility. Duplicates were removed, and all literature screening was independently performed by two reviewers (Yuhang Wu and Qingyu Du), with unresolved eligibility discrepancies adjudicated by a third senior author (Chenglin Zhao). This systematic and reproducible approach ensures the comprehensiveness and reliability of the evidence base for the present meta-analysis.

### Inclusion and exclusion criteria

The inclusion criteria for all the articles identified in the literature search were as follows: (1) patients who were histologically diagnosed with colorectal carcinoma or rectal carcinoma; (2) patients who were scheduled for elective colorectal surgery; and (3) only RCTs were included.

The exclusion criteria were as follows: (1) animal research; (2) non-original research (reviews, comments, letters and case reports); (3) duplicate records identified across different databases; (4) studies with incomplete, unextractable, or missing key outcome data; and (5) patients diagnosed with immune system diseases.

### Assessment of study quality

We screened studies that met the PRISMA (Preferred Reporting Items for Systematic Reviews and Meta-Analyses) guidelines. After that, the quality of the included studies was assessed using the version 2 of the Cochrane risk-of-bias tool for randomized trials (RoB 2), and two review authors independently evaluated the risk of bias across five domains: bias arising from the randomization process; bias due to deviations from intended interventions; bias due to missing outcome data; bias in measurement of the outcome; and bias in selection of the reported result Cochrane. Each domain was rated as “Low risk,” “Some concerns,” or “High risk” based on answers to structured signaling questions and the tool's embedded algorithms and Cochrane Methods.

### Statistical analysis

For dichotomous outcomes, including infectious complications, anastomotic leakage and ileus, the relevant effect measures were reported as odds ratios (ORs) with 95% confidence intervals (95%CIs). In contrast, weighted mean differences (WMDs) were employed to quantify the effect size for the continuous outcome of length of stay (LOS). The DerSimonian-Laird random-effects model was used to pool the effect sizes across the included studies. Statistical heterogeneity among the trials was assessed using the *I*^2^ statistic combined with the χ^2^ test (with *P* < 0.100 defined as the threshold for significant heterogeneity); an *I*^2^ value of 50% or greater was considered indicative of substantial heterogeneity. Additionally, odds ratios (ORs) and hazard ratios (HRs) with corresponding 95% CIs were calculated using fixed- or random-effects models to evaluate relevant clinical endpoints. In addition, outcomes such as complications and LOS were stratified into two subgroups according to the time of enteral immunonutrition administration (i.e., preoperative vs. postoperative), and subsequent subgroup analyses were performed following the aforementioned statistical methods. All the statistical analyses of this meta-analysis were performed using Stata statistical software (Version 10.0; StataCorp, College Station, TX, United States), with *P* < 0.05 set as the criterion for statistical significance.

## Results

### Characteristics of the studies included in the meta-analysis

Initially, we analyzed 131 studies, among which seven had duplicate data and 34 lacked full texts. Among the remaining 90 studies, 81 were excluded: 33 were review articles only, four were only animal studies, 25 in which the patients had other tumors, nine in which the outcomes did not meet the inclusion criteria, and 10 in which the data could not be merged.

**Figure 1:**
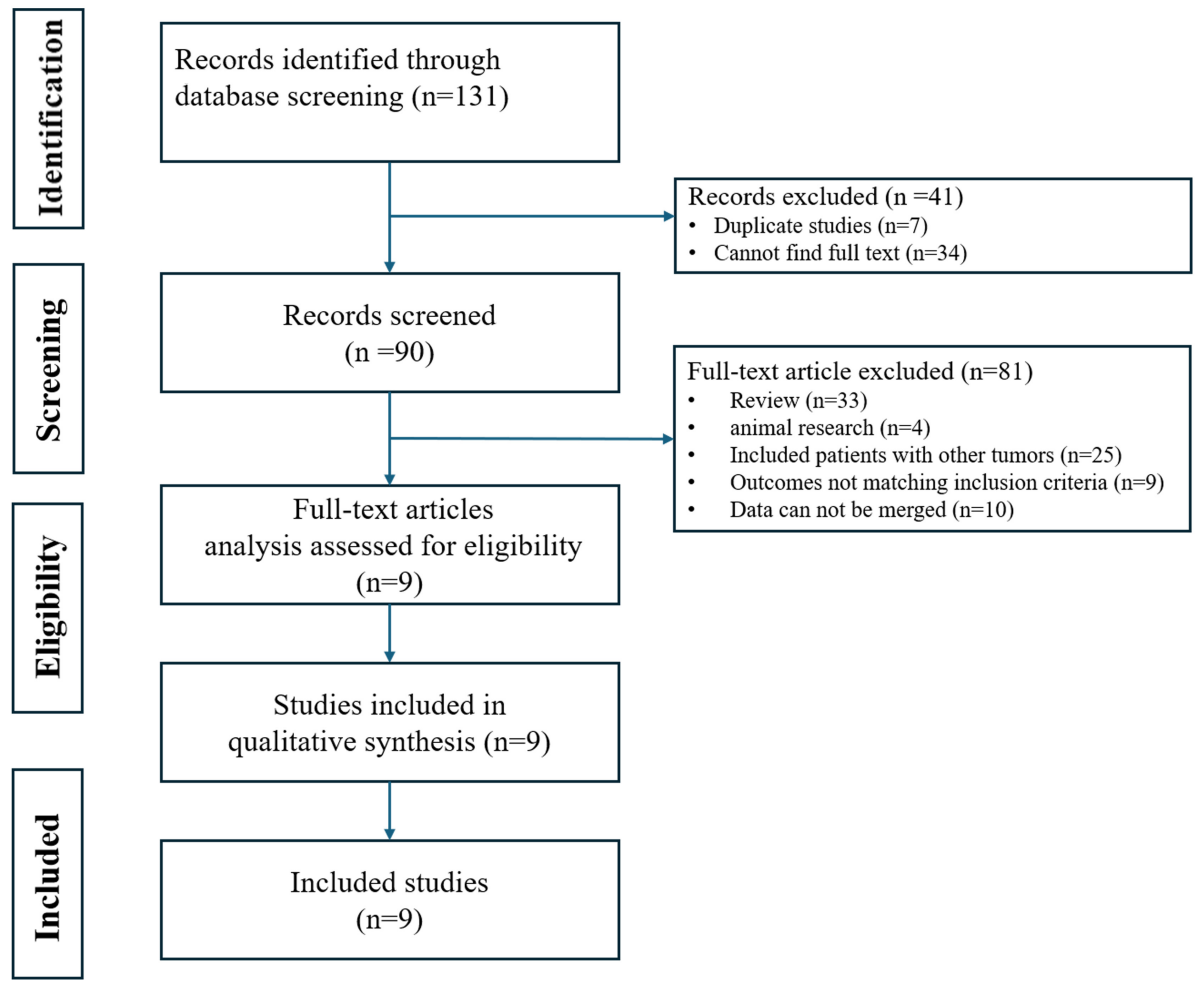
Flow diagram illustrating the process of selecting articles.

Nine articles were confirmed to be eligible for this meta-analysis. The risk of bias for all included randomized controlled trials was independently assessed by two reviewers using version 2 of the Cochrane risk-of-bias tool (RoB 2), with any disagreements resolved by discussion with a third reviewer. The overall methodological quality of the included studies was high. All the studies had a low risk of bias in terms of the randomization process, missing outcome data, outcome measurement, and selection of the reported result. Only a small number of studies raised some concerns regarding bias due to deviations from intended interventions, mainly owing to the nature of surgical and nutritional interventions, which made blinding difficult to implement. No study was judged to be at high risk of bias in any domain. A total of 1,198 patients were included in this meta-analysis (Figure 1, Table 1) (12–20).

**Table 1:** Main characteristics of all studies included in the meta-analysis.

Number	First author	Year	Country	Journal	Sample size	Intervention time	Nutritional status	Route of nutrition administration	NOS
1	Horie	2006	Japan	Surg Today	67	5 days before surgery	Well-nourished	Enteral nutrition	7
2	Moya	2016	Spain	Surg Endosc	122	Perioperative period	Well-nourished	Enteral nutrition	8
3	Tesauro	2021	Italy	Journal of Clinical Medicine	173	5 days before surgery	Well-nourished	Enteral nutrition	7
4	Moya	2016	Spain	Medicine	244	Perioperative period	Well-nourished	Enteral nutrition	9
5	Braga	2002	Italy	Surgery	200	5 days before surgery and perioperative period	Undifferentiated	Enteral nutrition	8
6	Finco	2007	Italy	Surg Endosc	28	Perioperative period	Undifferentiated	Enteral nutrition	6
7	Sun	2020	China	Hainan Medical Journal	60	5 days before surgery	Undifferentiated	Enteral nutrition	6
8	Jorge	2022	Spain	Nutrients	143	Postoperative period	Well-nourished	Enteral nutrition	7
9	Soo	2021	South Korea	Annals of Surgery	161	7 days before surgery	Well-nourished	Enteral nutrition	8

### Immunonutrition and infectious complications

**Figure 2:**
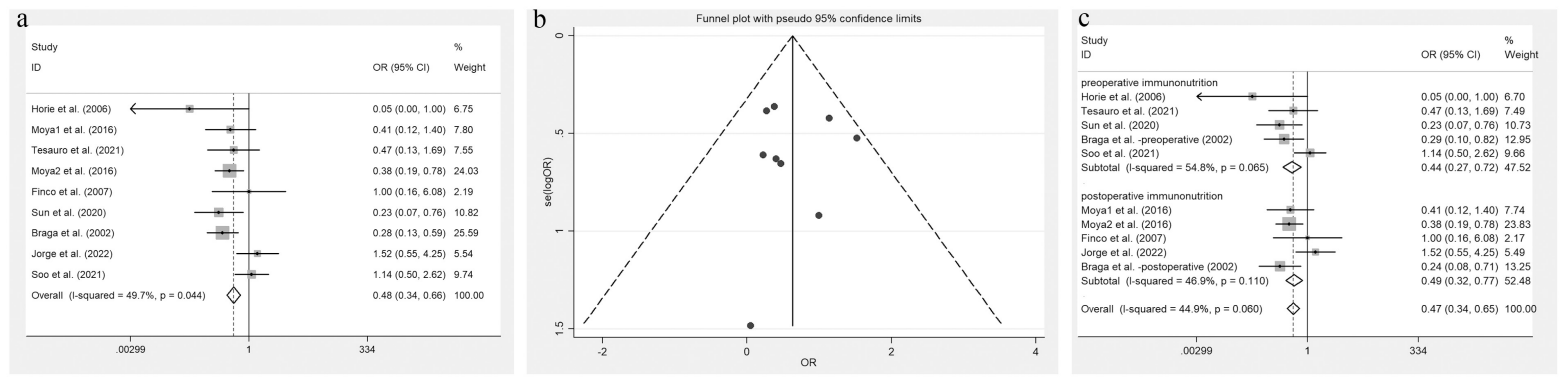
The plots illustrate the association between administering immunonutrition to patients and postoperative infectious complications. **(a)** forest plots, **(b)** funnel plots, **(c)** subgroup analyses for infectious complications according to intervention timing.

Herein, 1,198 CRC patients from nine studies were included, all of whom received enteral immunonutrition. A meta-analysis revealed that administering immunonutrition significantly reduced postoperative infectious complications (OR: 0.48; 95% CI: 0.34–0.66; *P* < 0.001), and moderate heterogeneity was detected across the included studies (*I*^2^ = 49.7%; *P* = 0.044). We performed Egger's test to assess potential publication bias. Egger's test (*P* = 0.464) revealed there was no significant evidence of publication bias (Figures 2a, b).

The results of the subgroup analysis according to immunonutrition timing (preoperative vs. postoperative) are shown in Figure 2c. Meta-regression showed that there was no significant difference of infection complications between preoperative and postoperative enteral immunonutrition subgroups (*P* = 0.647). According to the subgroup analysis, the risk of postoperative infectious complications was significantly lower in the preoperative enteral immunonutrition subgroup (OR: 0.51; 95% CI: 0.34 to 0.77; *P* = 0.001). Similarly, the postoperative enteral immunonutrition subgroup also exhibited a significant reduction in the risk of postoperative infectious complications (OR: 0.55; 95% CI: 0.37 to 0.80; *P* = 0.002).

### Immunonutrition and anastomotic leakage

**Figure 3:**
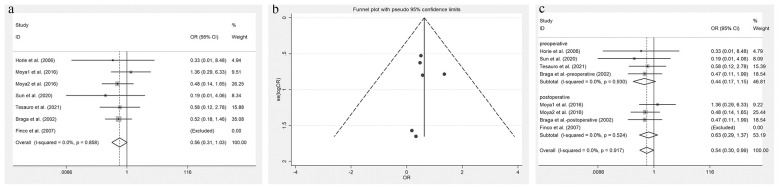
Results of the analysis between immunonutrition and anastomotic leakage. **(a)** forest plots, **(b)** funnel plots, **(c)** subgroup analysis for anastomotic leakage according to intervention timing.

Clinical data from 866 patients reported in six studies were included to analyze the relationship between enteral immunonutrition and anastomotic leakage. Postoperative anastomotic leakage in patients who underwent CRC surgery with enteral immunonutrition was lower than that in patients who underwent CRC surgery without enteral immunonutrition (OR: 0.56; 95% CI: 0.31–1.03; *P* = 0.063); however, the evidence is insufficient to confirm a statistically significant benefit, but a clinically important effect cannot be ruled out. There was no substantial heterogeneity across the included studies (*I*^2^ = 0.0%, *P* = 0.859). Moreover, we assessed potential publication bias using Egger's test. Egger's test revealed there was no significant evidence of publication bias (*P* = 0.550) (Figures 3a, b).

The results of the subgroup analyses for anastomotic leakage are presented in Figure 3c. Meta-regression showed there was no significant difference of the anastomotic leakage between preoperative and postoperative enteral immunonutrition subgroups (*P* = 0.655). In the subgroup analysis, the risk of anastomotic leakage did not demonstrate a significant decrease in the preoperative enteral immunonutrition subgroup (OR: 0.49; 95% CI: 0.21 to 1.16; *P* = 0.103). Likewise, the risk of anastomotic leakage was not significantly lower in the postoperative enteral immunonutrition subgroup (OR: 0.65; 95% CI: 0.32 to 1.34; *P* = 0.248).

### Immunonutrition and ileus

**Figure 4:**
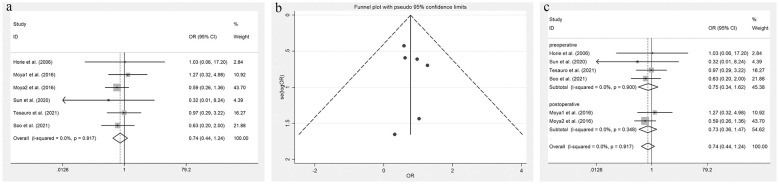
Results of the analysis between enteral immunonutrition and ileus. **(a)** forest plots, **(b)** funnel plots, **(c)** subgroup analyses for ileus according to intervention timing.

Six articles with a total of 827 CRC patients were included in this research to investigate the relationship between EN and ileus. We found no significant association between enteral immunonutrition and postoperative intestinal obstruction among CRC patients. (OR: 0.74; 95% CI: 0.44–1.24; *P* = 0.249), and heterogeneity was negligible (*I*^2^ = 0.0%; *P* = 0.917). We also evaluated potential publication bias using Egger's test. Egger's test revealed there was no significant evidence of publication bias (*P* = 0.825) (Figures 4a, b).

The results of the subgroup analysis for intestinal obstruction are presented in Figure 4c. Meta-regression showed that there was no significant difference of ileus between preoperative and postoperative enteral immunonutrition subgroups (*P* = 0.953). In the preoperative enteral immunonutrition subgroup, the risk of intestinal obstruction did not significantly decrease (OR: 0.76; 95% CI: 0.37–1.55; *P* = 0.455). The postoperative enteral immunonutrition subgroup also showed no significant reduction in intestinal obstruction risk (OR: 0.75; 95% CI: 0.40–1.42; *P* = 0.376).

### Immunonutrition and LOS

**Figure 5:**
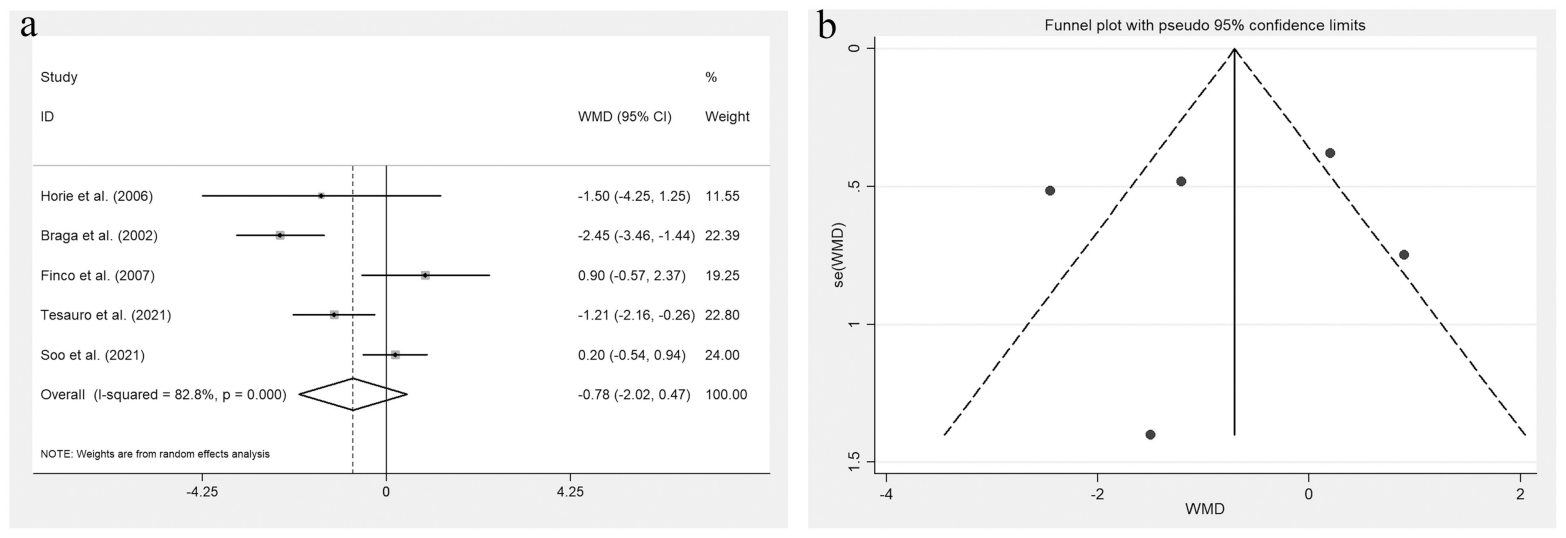
Results of the analysis between immunonutrition and LOS. **(a)** forest plots, **(b)** funnel plots.

With respect to LOS, five studies involving CRC patients reported an association between enteral immunonutrition and LOS. The pooled results revealed that there was no significant difference in the LOS between CRC patients who received enteral immunonutrition and those who did not receive enteral immunonutrition (WMD of −0.78; 95% CI: −2.02 to 0.47; *P* = 0.222). There was substantial between-study heterogeneity (*I*^2^ = 82.8%, *P* < 0.001). Furthermore, we performed Egger's test to assess potential publication bias, and the result showed that there was no significant evidence of publication bias (*P* = 0.842) (Figures 5a, b). Owing to the small number of included studies (*n* = 5) and the lack of data concerning the type of surgery, patient nutritional status, or geographic region, additional subgroup analyses were not performed to explore the sources of this heterogeneity.

## Discussion

While the progression of CRC is not as rapid as that of other cancers (21), its incidence rate is still extremely high (22), making it a major obstacle to increasing life expectancy. In China, the number of deaths attributed to CRC each year is countless. Faced with this challenge, surgery remains the only effective method for the radical cure of CRC to date (23). However, surgery undoubtedly imposes a significant physical burden on patients, especially elderly patients. Key indicators used to evaluate a patient's prognosis include the speed of postoperative recovery, the occurrence of complications, and the time to safe discharge from the hospital after treatment.

To reduce patient complications as much as possible, various approaches have been adopted. For example, intraperitoneal hyperthermic perfusion therapy (IPHP) is applied to decrease peritoneal seeding and metastasis (24), and preoperative antibiotic use helps lower the risk of postoperative infections (25). In recent years, many researchers have proposed immunonutrition therapy as a means to reduce patient complications (26, 27). Immunonutrients modulate the systemic inflammatory response, blunt pro-inflammatory cytokine release, and restore cellular immunity, which collectively mitigate infection risk and accelerate recovery; for instance, Xu et al. (26) confirmed that enteral immunonutrition can reduce postoperative infectious complications (particularly surgical site infections) and LOS in CRC patients, while preoperative immunonutrition (PIN) can restore cellular immune function and shorten the LOS. However, there is still controversy. For example, Finco et al. (12) reported that enteral immunonutrition could not lower the rate of postoperative infectious complications in surgical patients. Therefore, we performed a meta-analysis, which contrasted with the studies of Tesauro et al. and Finco et al., which reported a nonsignificant reduction in infectious complications with enteral immunonutrition. We concluded that both preoperative enteral immunonutrition and postoperative enteral immunonutrition reduce the risk of infections after surgery. However, we recommend immune-nutrition preparations before surgery.

With respect to postoperative anastomotic leak and ileus, Braga et al. (13) reported that enteral immunonutrition could significantly reduce the risk of postoperative anastomotic leak and ileus, whereas Horie et al. (14) and Moya et al. (15) failed to confirm a significant reduction in anastomotic leakage and ileus complications following enteral immunonutrition administration (17). Regarding the effect of enteral immunonutrition on the postoperative LOS of CRC patients, two studies yielded contradictory results: Tesauro et al. (16) reported that enteral immunonutrition could lower the LOS of CRC patients, but Moya et al. (15) reported that it could not lower that in their study. Our study did not find that preoperative immunonutrition significantly reduced the incidence of postoperative anastomotic leakage and intestinal obstruction in patients with colorectal cancer, and it also failed to demonstrate that preoperative immunonutrition could shorten the LOS in these patients. We performed a subgroup analysis by the timing of the intervention (preoperative vs. perioperative enteral immunonutrition). However, no significant difference was observed in the incidence of postoperative anastomotic leakage, intestinal obstruction, or LOS between patients who received preoperative enteral immunonutrition and those who received postoperative enteral immunonutrition.

This study had several limitations. First, although Akihisa et al. reported that a shift in the Th1/Th2 balance toward a Th2-dominant profile in the early postoperative stage is directly linked to the onset of such infectious complications following surgery (28), the way in which immunonutrition may improve postoperative outcomes in CRC patients remains unclear. Recent studies have preliminarily explored related mechanisms: omega-3 fatty acid-enriched immunonutrition can suppress proinflammatory cytokines (IL-6 and TNF-α) (29), remodel neutrophil leukotriene profiles (30), and reverse Th2 immune deviation while enhancing immune cell function (31). However, these findings are fragmented, and further mechanistic research is still needed. Second, several potential confounding factors that should be considered in the manuscript, such as variability in surgical technique, ERAS implementation, baseline nutritional status, and immunonutrition protocols, which may have influenced the observed outcomes. These potential confounding factors could cause publication bias. Therefore, publication bias cannot be ignored. Third, it should also address the potential for performance bias in the included RCTs, as the blinding of participants and personnel to a nutritional intervention is often impossible, which was noted in the risk-of-bias assessment. In addition, due to the small number of included studies and the lack of data on the type of surgery, patient nutritional status, or geographic region, additional subgroup analyses were not performed to explore the sources of this heterogeneity. Finally, different types and doses of key components of immunonutritional preparations (arginine, glutamine, and omega-3 fatty acids) were used by different authors. It is much better that a sensitivity analysis excluding studies that used formulations lacking a key component (e.g., no glutamine) would provide much more clinically useful information. Owing to the different types and doses of immunonutritional supplements for patients included in the literature not being the same and the lack of sufficient data for further analysis of the types and doses of key components of immunonutritional preparations in patients with CRC surgery, this study did not analyze the types and doses of immunonutritional supplements. We will explore the effects of different types and doses of immunonutritional supplements on CRC patients in subsequent studies.

## Conclusion

Our meta-analysis previously reported that administration of enteral immunonutrition is associated with a significant reduction in postoperative infectious complications in CRC patients. However, our study revealed that enteral immunonutrition is not associated with a reduced incidence of postoperative anastomotic leakage, ileus or LOS in patients with CRC. On the basis of our findings, we recommend the routine administration of immunonutritional supplements to CRC patients who have undergone surgery. Both preoperative and postoperative administration of immunonutritional supplements may significantly reduce the incidence of postoperative infection complications. Our results provide a theoretical basis for the clinical use of immunonutritional supplements in CRC surgery patients. These findings require much larger and higher-quality studies for further confirmation.

## Data Availability

The original contributions presented in the study are included in the article/supplementary material, further inquiries can be directed to the corresponding author.
